# The physics and technology of diagnostic ultrasound – a practitioner's guide

**DOI:** 10.1002/jmrs.10

**Published:** 2013-04-25

**Authors:** Ling Lee

**Affiliations:** Royal Brisbane and Women's Hospital, Queensland Health Butterfield StHerston, Brisbane, QLD 4029, Australia

The physics of ultrasound is the first module in most sonography courses and students often find this topic rather daunting. However, to be a qualified sonographer, it is essential that one understands the physics of ultrasound and is able to apply it on a daily basis as it underpins the fundamentals of diagnostic ultrasound. Dr. Robert Gill is well known for his expertise in the physics and technology of diagnostic ultrasound and has years of teaching experience in the field. His “practitioner's guide” on the topic is an excellent resource for both qualified and student sonographers. This book fills the needs of qualified sonographers to have a lightweight, easy reference manual to refresh their memory on practical concepts. It also serves as a suitable resource for students as a study guide for the physics component of the ultrasound examinations.

The book contains the essential knowledge on starting from the very basics of ultrasound physics, such as the formation of pulse, knobology, types of transducers, artefacts, principles of Doppler, and bioeffects of diagnostic ultrasound. It also covers advanced topics such as spatial compounding, harmonics, and 3D/4D ultrasound. Therefore, all the fundamental knowledge on the physics of diagnostic ultrasound, as required of the physics module of most Australian postgraduate diplomas of medical ultrasonography, are covered.

The content is laid out in a systematic manner that eases the student's learning process starting from the basic fundamentals to more advanced concepts over 12 comprehensive chapters. The main takeaways from each chapter are provided in concise summaries that are easily understood. Furthermore, the book is populated with sonograms and diagrams that facilitate the learning process of difficult abstract concepts such as formation of artefacts. I particularly like the effective use of colour printing for topics on Doppler principles and artefacts, as colour images allow a better appreciation and understanding of the sonograms obtained through color Doppler, power Doppler, and pulse-wave Doppler.

'As most students pursue the medical ultrasonography qualification several years after completing tertiary level physics, many find it difficult and confusing to perform calculations such as the Doppler shift. This book presents the mathematical and physics formulas in an easy-to-understand and informative manner in Chapter 1 to allow readers to refresh their memory. The chapter includes basic mathematical exercises such as rearranging algebraic equations, explaining scientific and mathematical notations such as natural logarithm and exponential functions, and more by preparing students from the very basics and building their foundation in basic algebra before introducing the more complex physics equations. This builds the students' confidence in tackling such a challenging area. With the correct answers to each of the questions at the back of the book, this allows students to quiz themselves and test their understanding independently.

The “suggested activities” at the end of each chapter encourages students to put into practice what they have learnt. It reduces the abstractness of the theoretical physics component as students often grasp applied concepts more easily. I encourage students to complete the “suggested activities” in a group setting as this improves the learning process and encourages further interest in more advanced topics and the pursuit of in-depth knowledge in ultrasound.

In conclusion, this is an excellent resource for both qualified sonographers and student sonographers. For the qualified sonographers, this book serves as a handbook for quick referencing or as a teaching tool. Being lightweight, well structured, and compact, it is very handy in a busy working environment. For student sonographers, it contains much of the fundamental knowledge required for them to pass their examinations and delivers the content in a clear and concise manner. I highly recommend the use of this practitioner's guide and certainly appreciate the time and effort of the author in publishing this manual that is of benefit to many professionals in the field of diagnostic ultrasound.

